# Endoscopy-Based Deep Convolutional Neural Network Predicts Response to Neoadjuvant Treatment for Locally Advanced Rectal Cancer

**DOI:** 10.3389/fphys.2022.880981

**Published:** 2022-04-27

**Authors:** Xijie Chen, Junguo Chen, Xiaosheng He, Liang Xu, Wei Liu, Dezheng Lin, Yuxuan Luo, Yue Feng, Lei Lian, Jiancong Hu, Ping Lan

**Affiliations:** ^1^ Guangdong Institute of Gastroenterology, Guangdong Provincial Key Laboratory of Colorectal and Pelvic Floor Diseases, The Sixth Affiliated Hospital, Sun Yat-sen University, Guangzhou, China; ^2^ Department of Gastrointestinal Surgery, The Sixth Affiliated Hospital, Sun Yat-sen University, Guangzhou, China; ^3^ Department of Colorectal Surgery, The Sixth Affiliated Hospital, Sun Yat-sen University, Guangzhou, China; ^4^ Department of Pathology, The Sixth Affiliated Hospital, Sun Yat-sen University, Guangzhou, China; ^5^ Tianjin Economic-Technological Development Area, Yujin Digestive Health Industry Research Institute, Tianjin, China; ^6^ Department of Endoscopic Surgery, The Sixth Affiliated Hospital, Sun Yat-sen University, Guangzhou, China; ^7^ Department of Network Management, The Sixth Affiliated Hospital, Sun Yat-sen University, Guangzhou, China

**Keywords:** treatment response, endoscopy, deep convolutional neural network, rectal cancer, neoadjuvant therapy

## Abstract

**Background and Aims:** Although the wait and watch (W&W) strategy is a treatment choice for locally advanced rectal cancer (LARC) patients who achieve clinical complete response (cCR) after neoadjuvant therapy (NT), the issue on consistency between cCR and pathological CR (pCR) remains unsettled. Herein, we aimed to develop a deep convolutional neural network (DCNN) model using endoscopic images of LARC patients after NT to distinguish tumor regression grade (TRG) 0 from non-TRG0, thus providing strength in identifying surgery candidates.

**Methods:** A total of 1000 LARC patients (6,939 endoscopic images) who underwent radical surgery after NT from April 2013 to April 2021 at the Sixth Affiliated Hospital, Sun Yat-sen University were retrospectively included in our study. Patients were divided into three cohorts in chronological order: the training set for constructing the model, the validation set, and the independent test set for validating its predictive capability. Besides, we compared the model’s performance with that of three endoscopists on a class-balanced, randomly selected subset of 20 patients’ LARC images (10 TRG0 patients with 70 images and 10 non-TRG0 patients with 72 images). The measures used to evaluate the efficacy for identifying TRG0 included overall accuracy, sensitivity, specificity, positive predictive value (PPV), negative predictive value (NPV), and area under the receiver operating characteristic curve (AUROC).

**Results:** There were 219 (21.9%) cases of TRG0 in the included patients. The constructed DCNN model in the training set obtained an excellent performance with good accuracy of 94.21%, specificity of 94.39%, NPV of 98.11%, and AUROC of 0.94. The validation set showed accuracy, specificity, NPV, and AUROC of 92.13%, 93.04%, 96.69%, and 0.95, respectively; the corresponding values in the independent set were 87.14%, 92.98%, 91.37%, and 0.77, respectively. In the reader study, the model outperformed the three experienced endoscopists with an AUROC of 0.85.

**Conclusions:** The proposed DCNN model achieved high specificity and NPV in detecting TRG0 LARC tumors after NT, with a better performance than experienced endoscopists. As a supplement to radiological images, this model may serve as a useful tool for identifying surgery candidates in LARC patients after NT.

## Introduction

The incorporation of neoadjuvant therapy (NT) can help downstage and downsize primary rectal cancer, prevent local recurrence, and increase the possibility of sphincter preservation (Maas et al., 2010; Al-Sukhni et al., 2016). In contrast, radical surgery is invasive and can lead to severe complications, including permanent stoma, sexual, bladder, and bowel dysfunction. The perioperative mortality rates of radical surgical resection of LARC are as high as 2–5% (Borowski et al., 2010; Marijnen, 2015). Hence, LARC patients who have a clinical complete response (cCR, highly suspected as pathological CR [pCR]) after NT sometimes opt for a wait and watch (W&W) strategy. Indeed, some studies have found that among LARC patients who had a cCR after NT, those who opted for a W&W strategy had the same survival rate as those who underwent radical surgery (Coraglio et al., 2020; Simpson et al., 2020).

However, there is no standardized definition of cCR (van der Valk et al., 2018). High rates of residual tumor cells have been found in tumor specimens resected from cCR patients, which indicates poor consistency between cCR and pCR (Hiotis et al., 2002; Guillem et al., 2005). Among patients with cCR but not pCR who opt for the W&W strategy, a burst local recurrence within 2 years followed by difficult salvage surgery with more complex complications are foreseeable problems. Thus, current evidence suggests that the W&W strategy should be applied with caution.

Deep convolutional neural network (DCNN), a branch of artificial intelligence (AI), has a unique capacity for the integration of high-dimensional data, and is well suited to the medical field, with promising applications in capturing the features of deep layers (Li et al., 2018; Xie et al., 2020; Jiang et al., 2021). The DCNN network mimics the structure and activity of the brain neurons, which is logically in line with human thinking, and optimized on this basis. A DCNN model enables machines to train various given images derived from different inspection equipment and extracts specific clinical characteristics using a backpropagation algorithm. Based on these clinical characteristics, the machine is able to make diagnosis from newly acquired clinical images prospectively. Few studies have investigated the utility of DCNNs for the analysis of endoscopic images to detect TRG. The present study mainly aimed to develop a DCNN model to evaluate TRG0 using endoscopic images in LARC patients after NT, and compare the model’s performance with that of experienced gastrointestinal endoscopists on the same test set. In this way, we hoped to avoid the incorrect application of the W&W strategy, which would result in patients missing their optimal time window for surgery.

## Materials and Methods

### Ethics Statement

This study was conducted in concordance with the ethical standards of the World Medical Association, the tenets of the Declaration of Helsinki, and the Ethical Guidelines for Clinical Research. In addition, the study was approved by the institutional review board of the Sixth Affiliated Hospital, Sun Yat-sen University (no. 2021ZSLYEC-063). Informed consent was not required because pre-existing data were used.

### Patient Selection

We initially evaluated 1,103 consecutive patients who were pathologically diagnosed with rectal cancer. All the patients underwent radical surgery after NT between April 2013 and April 2021 in the Sixth Affiliated Hospital, Sun Yat-sen University.

Patients who received neoadjuvant chemoradiotherapy were given long-course radiotherapy of 50 Gy in 25 fractions or short-course radiotherapy of 5 Gy once a day for 5 days to the clinical target volume. The concurrent chemotherapy regimen was mainly based on oral/intravenous 5-fluorouracil, or combined with oxaliplatin/irinotecan. Patients treated with neoadjuvant chemotherapy alone shared the same chemotherapy regimen with the former.

The inclusion criteria were as follows: 1) patients, primary T3-4/N+ rectal cancer with post-treatment restaging endoscopy data stored in our center, 2) intervention, NT followed by radical surgery, and 3) outcomes, tumor regression grade (TRG) assessments (derived from surgical pathology reports) available. We excluded patients who had 1) ambiguous endoscopy images (e.g., low-resolution, under-focus, etc.), 2) bleeding on the tumor surface by endoscopic procedure or inadequate bowel preparation (Boston Bowel Preparation Score lower than 2 for the examined regions of the rectum), which blurs the tumor surface and hinders feature extraction, 3) endoscopic imaging with operating forceps, 4) insufficient NT courses (withdrawal from planned chemoradiotherapy protocol), 5) simultaneous colorectal cancer, 6) familial adenomatous polyps, Lynch syndrome, ulcerative colitis, or other diseases with genetic susceptibility to colorectal cancer, or 7) missing data. The raw screening process of the images was evaluated by two experienced endoscopists; when disagreement occurred, the opinion of the third senior investigator was requested. To create a more homogeneous model, at least four images which met the prespecified requirements for image quality of each person were required.

### Data Preparation

Preoperative, unamplified, white-light endoscopic images with diagnostic reports and postsurgical pathology reports were collected from the digital image acquisition and pathology report systems, respectively. Restaging endoscopic images were collected 6–8 weeks after finishing NT. The interval time from finishing NT to surgery was described as 6–12 weeks. We extracted tumor information such as differentiation and TRG. As described by the AJCC 8th edition staging system (Weiser, 2018), TRGs were defined as follows: TRG0 (complete response), no viable cancer cells; TRG1 (moderate response), single or small groups of cancer cells; TRG2 (minimal response), residual cancer outgrown by fibrosis; and TRG3 (poor response), minimal or no tumor kill and extensive residual cancer. Patients’ demographic information, including sex, age, and tumor biomarkers, was acquired from electronic or paper health records.

### Outcome and Group Assignment

The primary outcome of this study was to develop a DCNN model to discriminate TRG0 from non-TRG0 (TRG1—TRG3) among LARC patients who had received NT. We randomly divided patients treated between April 2013 and October 2020 into a training set and a validation set at a ratio of 9:1; the training set was used for constructing the DCNN model, while the validation set was used to examine its predicting capability. Patients treated between November 2020 and April 2021 were assigned to a completely independent test set to externally evaluate the model’s performance in tumor response prediction. Receiver operating characteristic (ROC) curves were plotted to detect the discriminative power, which was quantified using the area under the ROC curve (AUROC).

To compare the performance of DCNN model with that of veteran endoscopists, we did a manual review study (reader study) in which three endoscopists reviewed the same test set of 20 patients’ LARC images after NT (10 TRG0 patients with 70 images and 10 non TRG-0 patients with 72 images), which were class-balanced and randomly selected from the independent test set. Each endoscopist had more than 3 years of work experience and performed over 5,000 endoscopy examinations.

### DCNN Model Construction

**FIGURE 1:**
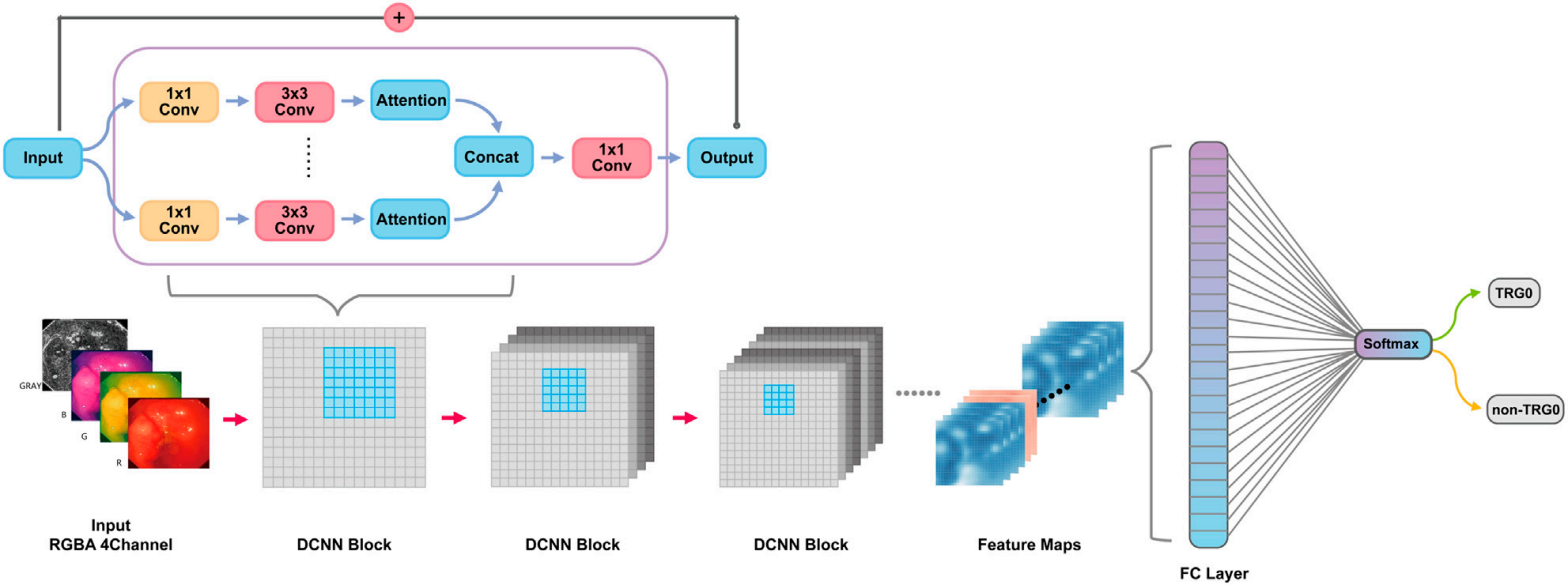
The key architecture diagram of the DCNN model. DCNN, deep convolutional neural network.

**FIGURE 2:**
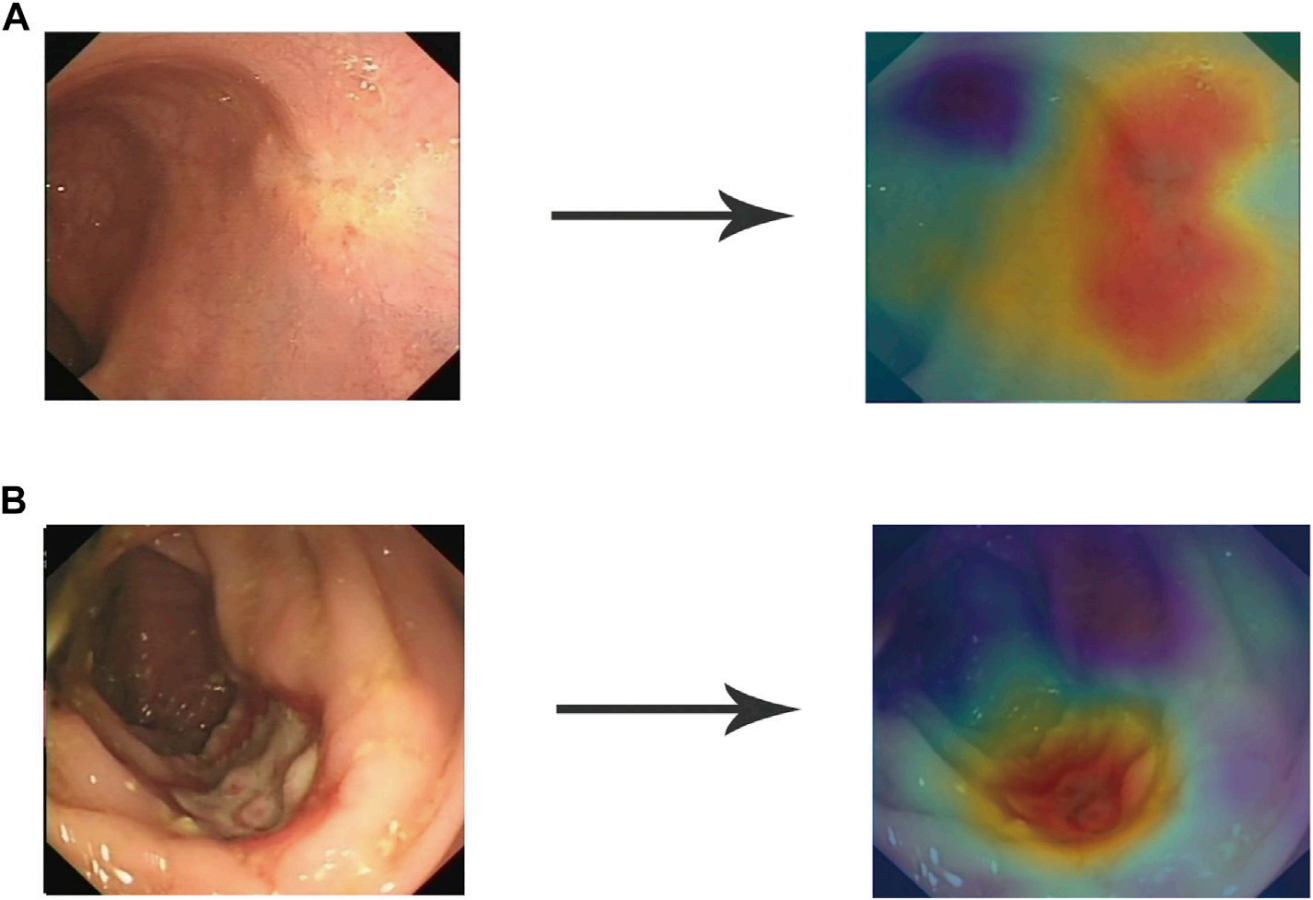
Heat map to visualize the DCNN model. The heat map is mainly composed of red tones and blue tones. The red tones reveal the very region of the input image that activates the category (TRG0 or non-TRG0), which is what we are interested in, while the blue tones are the regions of non-interest. The darker the red tone, the more important the region. **(A)** Representative image of TRG0. **(B)** Representative image of non-TRG0. DCNN, deep convolutional neural network.

After image preprocessing (details shown in the supplementary method section, Supplementary Figure S1), we tailored a modification of the ResNeSt-50 variant, an existing high-performance neural network, and preprocessed the pathological data to obtain more effective features. DCNN is famous for its powerful ability of feature extraction and classification and recognition. In this study, DCNN network named ResNeSt-50 was used to identify endoscopic images as TRG0 or non-TRG0. We overlayed the RGB three channels of the image with the gray image with enhanced edge features, and synthesize four channels as input information. DCNN used each internal block to complete feature extraction, and continuously upgraded the dimension in this process to extract some abstract high-level features, and finally form several feature maps. At the end of DCNN, these feature maps would be transformed into a full connection layer (FC layer), and the regression operation would be carried out by using softmax function to obtain the results of TRG0 or non-TRG0 (Figure 1). To visualize the characteristics of the lesions, we created a heat map (Figure 2) to better display the features, making the network more sensitive to the local details of the space and image. We used label smoothing technology to prevent overfitting, thereby increasing the fault tolerance rate, generating a better calibration network, and ultimately incubating a more accurate prediction model on invisible data. In addition, a weighting method was used to eliminate the bias caused by imbalanced data distribution; the formula was as follows: category weight = (total sample–current category samples)/total sample. Finally, after pre-training by the ImageNet, we used our data for fine-tuned learning and model training. The initial learning rate was 1e–4, and the momentum was 0.9; we stopped the training at 300 epochs.

### Evaluation Indicators and Statistical Analysis

Evaluation indicators, including AUROC, accuracy, sensitivity, specificity, positive predictive value (PPV), and negative predictive value (NPV). The 95% confidence interval (CI) was calculated using a two-sided exact binomial test using the Clopper-Pearson interval. For clinicopathological data, continuous random variables with normal distributions were presented as mean and standard deviation, and non-normally distributed variables were presented as median and interquartile range. Categorical variables were presented as frequencies and percentages. For the sake of actual clinical practice, continuous random variables were transformed into categorical variables when appropriate. The chi-square test was used for qualitative data. A stepwise binary logistic regression was performed to identify factors that were independently associated with our outcome of interest. The Pytorch (version 1.7.1) deep learning platform was employed for training and validating our DCNN model. For the reader study, TRG0 prediction agreement among the endoscopists was calculated using Fleiss’ kappa. All the analyses were carried out using Python (version 3.7, Python Software Foundation, Wilmington, DE) and IBM SPSS statistics (version 26.0, IBM Corp., New York, USA). A two-sided *p* value of less than 0.05 was deemed statistically significant.

## Results

### Patient Selection and Demographic Characteristics

**FIGURE 3:**
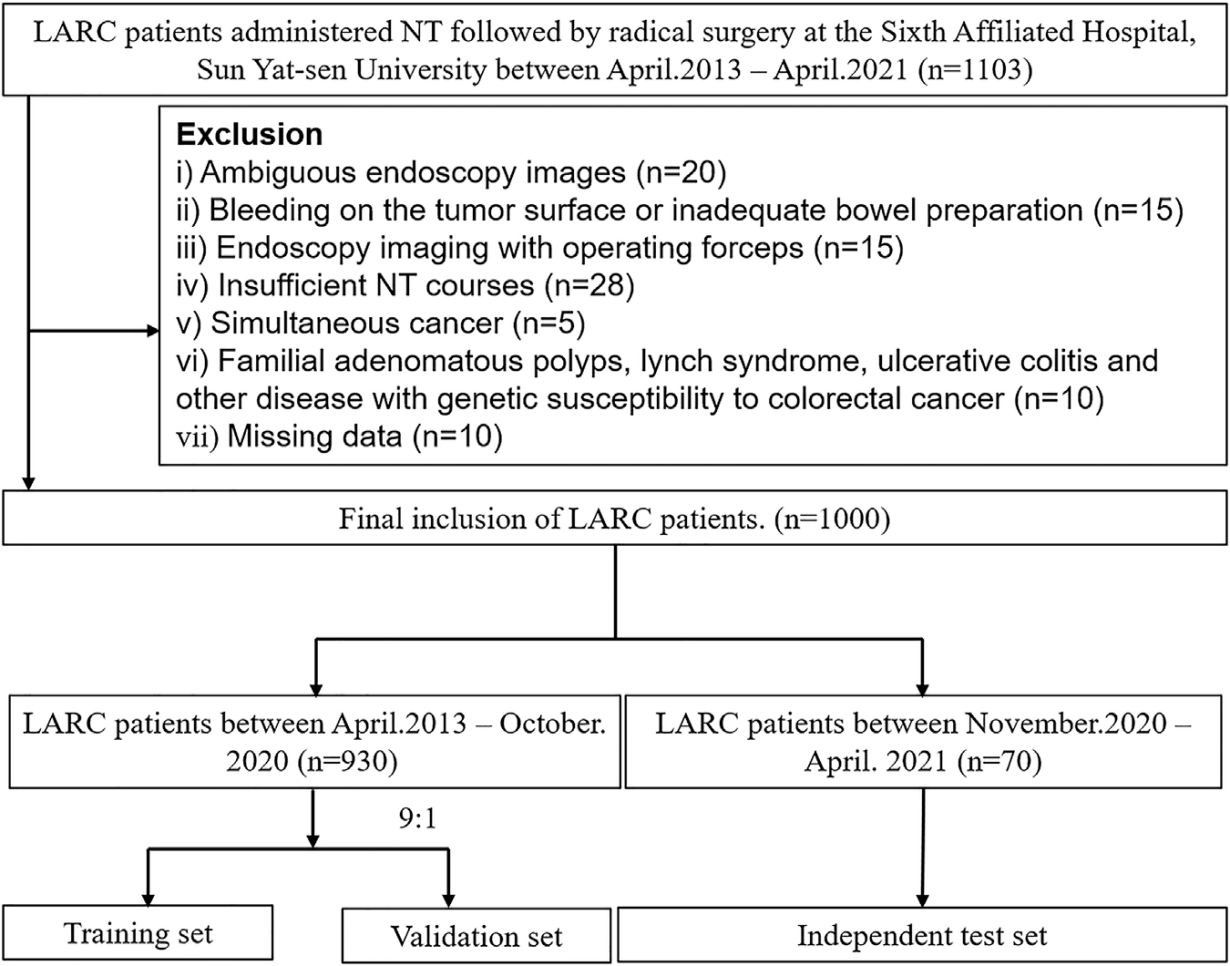
Flow chart of the study. LARC, locally advanced rectal cancer; NT, neoadjuvant therapy.

A total of 1,000 LARC patients with 6,939 images obtained between April 2013 and April 2021 were included in this study (Figure 3). Among them, 219 patients had TRG0, accounting for 21.9% of the total population. The training and validation sets included 930 patients (6,500 endoscopic images), of whom 206 patients (1,433 images) were TRG0, and 724 patients (5,067 images) were non- TRG0. The independent set comprised 70 patients (439 images), among which 13 (18.6%) patients with 83 images were TRG0, and 57 patients (356 images) were non- TRG0. The characteristics of the two cohorts were comparable. The median age of the patients was 57 years, and male patients accounted for majority of subjects ( ∼ 70%). All patients received neoadjuvant chemotherapy, and >40% of patients were administered neoadjuvant radiotherapy. Most of the cancers were moderately or poorly differentiated, and located in the middle or lower rectum. Obvious tumor regression could be observed in terms of the T/N stage and carcinoembryonic antigen (CEA) level after NT (Table 1). And we found that patients who were less than 50 years old, had received neoadjuvant radiotherapy, had well-differentiated tumors, or had negative preoperative serum CEA levels (<5 μg/ml) were more likely to achieve TRG0. (Supplementary result section, Supplementary Table S1).

**TABLE 1:** Characteristics of locally advanced rectal cancer patients.

	Training Set + Validation Set	Independent Test Set	*p*
TRG	—	—	0.64
0	206 (22.2%)	13 (18.6%)	—
1	200 (21.5%)	12 (17.1%)	—
2	415 (44.6%)	36 (51.4%)	—
3	109 (11.7%)	9 (12.9%)	—
Age	57 (47–64)	57.5 (50–64)	0.73
Sex	—	—	0.20
male	669 (71.9%)	47 (67.1)	—
female	261 (28.1%)	12 (32.9)	—
BMI	22.6 (20.5–24.8)	22.6 (20.8–24.1)	0.76
Neoadjuvant chemotherapy	—	—	1
yes	930 (100%)	70 (100%)	—
no	0 (0)	0 (0)	—
Neoadjuvant radiotherapy	—	—	0.74
yes	380 (40.9%)	30 (42.9%)	—
no	550 (59.1%)	40 (57.1%)	—
Differentiation	—	—	0.36
well	259 (27.8%)	14 (20.0%)	—
moderate	592 (63.7%)	49 (70.0%)	—
poor	79 (8.5%)	7 (10.0%)	—
Pre-T^a^	—	—	0.01^b^
2	34 (3.7%)	0 (0)	—
3	573 (61.6%)	36 (51.4%)	—
4	180 (19.4%)	22 (31.4%)	—
Pre-N^a^	—	—	0.25
0	158 (17.0%)	17 (24.3%)	—
1	297 (31.9%)	18 (25.7%)	—
2	315 (34.0%)	23 (32.8%)	—
ypT	—	—	0.56
0	208 (22.4%)	13 (18.6%)	—
1	67 (7.2%)	5 (7.1%)	—
2	223 (24.0%)	17 (24.3%)	—
3	417 (44.8%)	35 (50.0%)	—
4	15 (1.6%)	0 (0%)	—
ypN	—	—	0.56
0	706 (75.9%)	57 (81.4%)	—
1	158 (17.0%)	9 (12.9%)	—
2	66 (7.1%)	4 (5.7%)	—
initial CEA	4.5 (2.4–10.5)	5.0 (2.5–12.0)	0.26
preoperative CEA	2.78 (1.90–4.71)	2.4 (1.7–3.2)	0.73
Distal margin from the anal verge/mm^b^	52 (36–72)	50 (32–65.8)	0.17

a Incomplete data.

b Significant different.

TRG, tumor response grade; BMI, body mass index; pre-T, Pretreatment T stage.

Pre-N, Pretreatment N stage; CEA, carcinoembryonic antigen.

### Modified DCNN Model Showed Good Performance in Predicting TRG Classification

**FIGURE 4:**
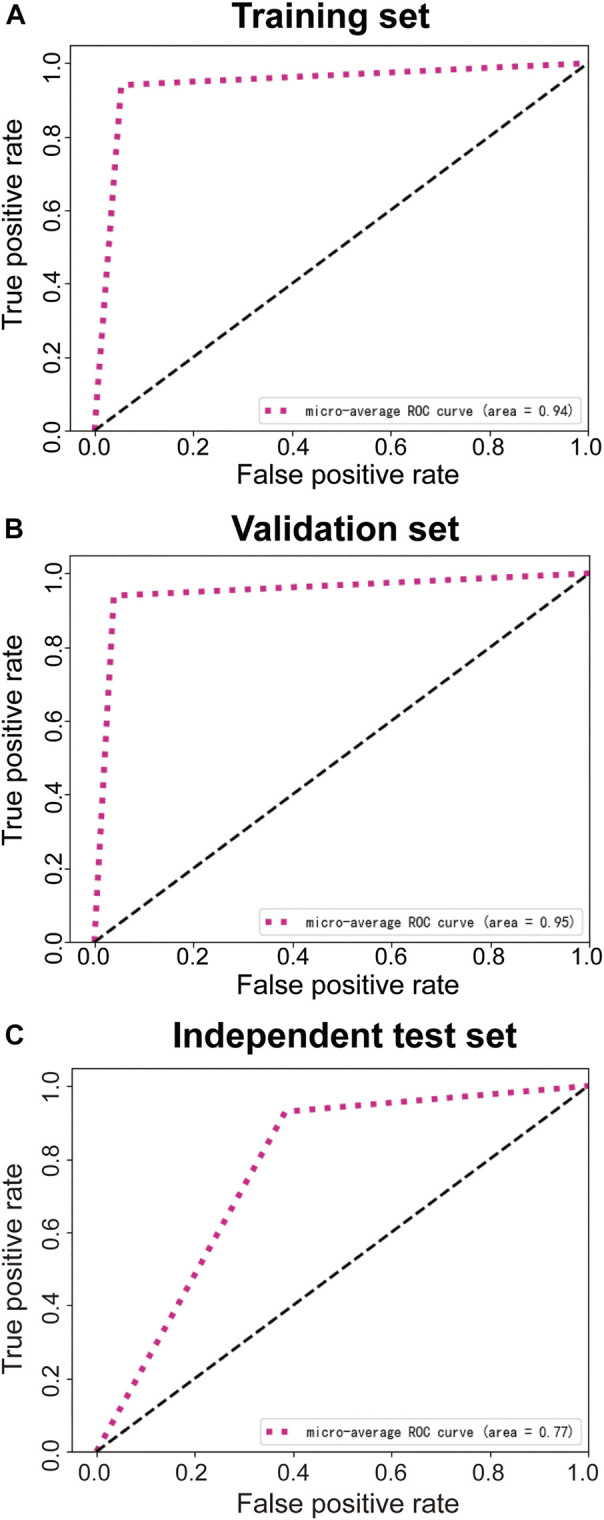
ROC curves of the training set **(A)**, validation set **(B)**, and independent test set **(C)**. ROC, receiver operating characteristic.

Using the modified ResNeSt-50 variant, we constructed a DCNN model classifier to accurately predict tumor response in LARC patients who had received NT. In the training cohort, the model successfully recognized 4,308 images for non-TRG0 and 1,205 images for TRG0, with 83 images misdiagnosed as non-TRG0 and 256 images misdiagnosed as TRG0, yielding an AUROC of 0.94 (95% CI: 0.93, 0.95), suggesting no departure from the perfect fit (Figure 4A), and the overall accuracy of the model was 94.21% (95% CI: 0.94, 0.95), with a sensitivity, specificity, PPV, and NPV of 93.56% (95% CI: 0.92, 0.95), 94.39% (95% CI: 0.94, 0.95), 82.48% (95% CI: 0.80, 0.84), and 98.11% (95% CI: 0.98, 0.99), respectively. In the validation cohort, the model successfully recognized 468 images for non-TRG0 and 129 images for TRG0; with 16 images misdiagnosed as non-TRG0 and 35 images misdiagnosed as TRG0, yielding an AUROC of 0.95 (95% CI: 0.92, 0.98) (Figure 4B), and the overall accuracy was 92.13% (95% CI: 0.90, 0.94), with a sensitivity, specificity, PPV, and NPV of 88.97% (95% CI: 0.83, 0.93), 93.04% (95% CI: 0.90, 0.95), 78.66% (95% CI: 0.72, 0.84), and 96.70% (95% CI: 0.95, 0.98), respectively. In the independent test set, the model successfully recognized 53 patients for non-TRG0 and 8 patients for TRG0, with 4 patients misdiagnosed as non-TRG0 and 5 patients misdiagnosed as TRG0, yielding an AUROC of 0.77 (95% CI: 0.65, 0.93) (Figure 4C), and the overall accuracy was 87.14% (95% CI: 0.76, 0.94), with a sensitivity, specificity, PPV, and NPV of 61.53% (95% CI: 0.32, 0.85), 92.98% (95% CI: 0.82, 0.98), 66.67% (95% CI: 0.35, 0.89), and 91.37% (95% CI: 0.80, 0.97), respectively, indicating perfect reproducibility of the DCNN model (Table 2). We tested the performance of the DCNN model by using Chi-square and univariate logistic regression methods, and both showed that the model predicted actual events well (Table 3).

**TABLE 2:** Efficacy of the DCCN model.

	Sensitivity	Specificity	PPV	NPV	Accuracy	AUROC
Training set	93.56% (95% CI: 0.92, 0.95)	94.39% (95% CI: 0.94, 0.95)	82.48% (95% CI: 0.80, 0.84)	98.11% (95% CI: 0.98, 0.99)	94.21% (95% CI: 0.94, 0.95)	0.94 (95% CI: 0.93, 0.95)
Validation set	88.97% (95% CI: 0.83, 0.93)	93.04% (95% CI: 0.90, 0.95)	78.66% (95% CI: 0.72, 0.84)	96.69% (95% CI: 0.95, 0.98)	92.13% (95% CI: 0.90, 0.94)	0.95 (95% CI: 0.92, 0.98)
Independent test set	61.53% (95% CI: 0.32, 0.85)	92.98% (95% CI: 0.82, 0.98)	66.67% (95% CI: 0.35, 0.89)	91.37% (95% CI: 0.80, 0.97)	87.14% (95% CI: 0.76, 0.94)	0.77 (95% CI: 0.65, 0.93)

CI, confidence interval.

DCNN, deep convolutional neural network.

**TABLE 3:** Correlation of the DCNN model and actual events.

	Chi-Square		Univariate Logistic Regression		
	*χ2*	*P*	OR	95% CI	*P*
DCNN model (training set)	3876.33	<0.01^a^	167.07	132.08–211.32	<0.01^a^
Validation set	388.48	<0.01^a^	108.62	56.72–208.04	<0.01^a^
Independent test set	24.19	<0.01^a^	0.04	0.01–0.19	<0.01^a^

a Significant different.

DCNN, deep convolutional neural network.

**FIGURE 5:**
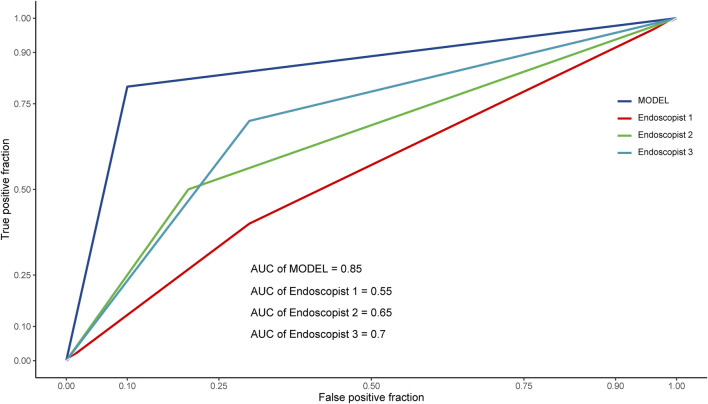
ROC curves of the reader study. ROC, receiver operating characteristic; AUC (AUROC), area under the receiver operating characteristic curve.

For the reader study, the model successfully recognized 9 patients for non-TRG0 and 8 patients for TRG0, with 2 patients misdiagnosed as non-TRG0 and 1 patient misdiagnosed as TRG0. Thus, the DCNN model achieved an accuracy of 85% (95% CI: 0.64-0.95), sensitivity of 80% (95% CI: 0.49-0.94), specificity of 90% (95% CI: 0.60-0.98), PPV of 88.89% (95% CI: 0.57-0.98), NPV of 81.82% (95% CI: 0.52-0.95), and AUROC of 0.85 (95% CI: 0.68-1). The Fleiss’ kappa value was 0.722 (*p* < 0.01), which showed significant inter- and intra-observer variability among experts on the assessment of TRG status based on the colonoscopic images. However, the DCNN model performed significantly better than the three endoscopists almost in all evaluating indicators (Table 4), and the performance of all three endoscopists was below the model’s ROC (Figure 5).

**TABLE 4:** Reader study.

	Sensitivity	Specificity	PPV	NPV	Accuracy	AUROC
DCNN model	80% (95% CI: 0.49, 0.94)	90% (95% CI: 0.60, 0.98)	88.89% (95% CI: 0.57, 0.98)	81.82% (95% CI: 0.52, 0.95)	85% (95% CI: 0.64, 0.95)	0.85 (95% CI: 0.69, 1)
Endoscopist 1	40% (95% CI: 0.17, 0.69)	70% (95% CI: 0.40, 0.89)	57% (95% CI: 0.25, 0.84)	53.8% (95% CI: 0.29, 0.77)	55% (95% CI: 0.34, 0.74)	0.55 (95% CI: 0.33, 0.77)
Endoscopist 3	80% (95% CI: 0.49, 0.94)	80% (95% CI: 0.49, 0.94)	80% (95% CI: 0.49, 0.94)	80% (95% CI: 0.49, 0.94)	80% (95% CI: 0.58, 0.92)	0.70 (95% CI: 0.49, 0.91)
Endoscopist 2	50% (95% CI: 0.24, 0.76)	80% (95% CI: 0.49, 0.94)	71.4% (95% CI: 0.36, 0.92)	61.5% (95% CI: 0.36, 0.82)	65% (95% CI: 0.43, 0.82)	0.65 (95% CI: 0.44, 0.86)

CI, confidence interval.

## Discussion

To the best of our knowledge, the present study is the first to develop an AI model by using endoscopic images from LARC patients after NT for the prediction of TRG0. Our DCNN model achieved good accuracy, high sensitivity, specificity, and NPV, and was proved to outperform experienced endoscopists. This model has the potential to serve as a robust supplementary tool to radiographic examinations for precisely selecting surgery candidates for LARC patients after NT.

NT followed by radical surgery is the standard of care for LARC. However, the fibrosis in the rectum and surrounding mesorectal tissue induced by NT increases the difficulty of radical surgery. Moreover, radical surgery is associated with mortality and morbidity such as permanent stoma, and can be physically, mentally, and emotionally traumatic to patients (Hupkens et al., 2017). Among LARC patients who achieve cCR, the W&W strategy or surgery could be chosen through the “share-decision making” policy in case of notifying specific risks. Some studies indicated that the prognosis of those who request the W&W strategy has not been found to be inferior to the prognosis of those who undergo radical surgery, and the absence of surgery greatly improves their quality of life (Sauer et al., 2004; Roh et al., 2009; Sebag-Montefiore et al., 2009; Valentini et al., 2011; Ortholan et al., 2012; Valentini et al., 2015). However, no standard criteria for cCR are available, cCR rates vary greatly from 10 to 78%, while the ensuing local recurrence or distant metastasis rates range from 7 to 33% (van der Valk et al., 2018; Pang et al., 2019; Asoglu et al., 2020; Pinto et al., 2020). This is partially attributable to an inflated false-positive rate due to estimations based on different standards. Therefore, enrollment in the W&W strategy without establishing proper surveillance protocols and salvage management might result in dismal outcomes. The current study on the DCNN model aimed to accurately identify candidates for surgery and reduce the unsuitable application of watchful waiting.

Computerized tomography (CT), MRI, ultrasonography, digital rectal examination, biopsy examination, and assessment of certain morphological features on endoscopy have been recommended as modalities for tumor-response assessment during NT (Glynne-Jones et al., 2017; Dattani et al., 2018; van der Valk et al., 2018), but none of them can precisely differentiate between patients who require watchful waiting and those who require radical surgery. Liu et al. (Liu et al., 2018) assessed 124 LARC patients, and found that only 25% sensitivity could be achieved regardless of the modality used to assess tumor response after NT, namely, MRI, ultrasonography, and endoscopic mucosal integrity. Although these techniques were associated with specificities of >90%, their use resulted in the recommendation of unnecessary surgery for at least 75% of pCR patients. Although some clinical characteristics were reported to be useful TRG0 predictors, there were significant inter-observer variability among three endoscopists in the reader study. Similarly, a study by van der Sande et al. (van der Sande et al., 2021) investigating the utility of endoscopic findings for TRG evaluation found that a flat scar was the feature most predictive of CR, with a PPV of 70–80%. However, due to the subjective nature of visual observation, only poor-to-moderate inter-observer agreement could be achieved among endoscopists. The potential of computer-aided systems to assist clinicians in diagnosing and evaluating gastrointestinal tumor lesions would help to deal with human subjectivity. Our findings suggest that the present deep learning model could provide added value as an automated screening tool of patient triage for confirmatory testing.

Recently, several radiomics and AI studies based on MRI have been conducted (Tang et al., 2019; Zhang et al., 2020). However, no radiomics model has yet been used in clinical practice. Factors that hamper the clinical application of radiomics are the lack of a unified standard for feature extraction and lack of evidence for the generalizability of the models across different MR scanners and different magnetic field strengths. In contrast, we constructed a DCNN model to predict TRG0 by using endoscopic images. The TRG0 rates in the training, validation, and independent test sets were 22.2, 20.4, and 18.6%, respectively, which are consistent with previous studies (Dattani et al., 2018; van der Valk et al., 2018). When developing this model, we focused on whether its sensitivity, specificity, and NPV were high enough to identify patients who indeed required surgery and to maximally recognize TRG0. As a result, although the diagnostic accuracy of the model may be affected due to various quality of optical imaging in different time periods as we divided the patients into three cohorts in chronological order, the model in the independent test set still accurately identified 8 CR and 53 non-CR patients; among the remaining patients, 5 were misdiagnosed with CR, and 4 were misdiagnosed with non-CR, yielding an accuracy of 87.14%, a specificity of 92.98%, and NPV of 91.37%. This means that if our DCNN model recommends surgery for a patient, we have over 91% confidence to make this decision, with a narrow false-negative rate of mistaking TRG0 patients as non-TRG0 patients. Generally, the DCNN model was superior to the other models mentioned above; it both reduced unnecessary watchful waiting and avoided missing the optimal time window for surgery as well as controlled the false-positive rate within an acceptable range. Last but not the least, unlike MRI-based predictive models, our DCNN model can be easily applied using any standard endoscopic system. Imaging that there is an AI module linkage endoscopic equipment, when an LARC patient undergoes an endoscopy, the AI module would capture tumor lesions automatically and calculate the probabilities of TRG0 in real time. A result of TRG0 would receive a W&W strategy recommendation if the lymph nodes are radiologically negative in the dialog box, otherwise a surgery advice. Sometimes we may find residual lesions in the intestinal lumen even it shows TRG0, and a transanal excision or endoscopic excision is warranted.

Several limitations of the present study should be noted. First, this study only assessed the local luminal tumor regression grade, and did not analyze lymph node involvement or distant metastasis status. However, it has been reported that positive lymph nodes are seldom found among patients with TRG0 (Debove et al., 2016). A multimodality DCNN model comprising MRI, endoscopy images, and clinicopathological characteristics is expected to overcome this limitation. Second, as this was a retrospective study, selection bias could not be avoided; however, the present study has the largest sample size among related studies. We included a total of 1,000 patients in our study, and performed image augmentation to further expand the sample quantity. Last, our model was based on a single-center study, and has not been validated by other centers, so our results should be interpreted with caution. Despite these limitations, a DCNN algorithm based on colonoscopy images could accurately reflect heterogeneity within the tumor, and the model was not affected by population distribution, making it possible to include more patients to improve accuracy. Further prospective multi-center research studies may improve the performance of our model, and efforts to raise model interpretability (e.g., by incorporating a visual representation of the network’s output) might help to increase trust in deep learning models.

In conclusion, the proposed DCNN model achieved high accuracy, sensitivity, specificity, and NPV in predicting TRG0 in LARC patients after NT, with a better performance than experienced endoscopists. This tool may serve as an ideal alternative method for monitoring treatment response during NT and could add value in identifying surgery candidates.

## Data Availability

The endoscopic images and clinicopathological data are not publicly available for patient privacy protection purposes but are available from the corresponding authors (LL, JH and PL) with a signed data access agreement. The deep convolutional neural network model was developed and improved based on the standard library in open-source platforms. The improved part was saved in our institute and can be obtained from our institute if necessary. Custom codes for the deployment of the model are available https://github.com/zhanghang1989/ResNeSt.
