# Supplementary material for: Endoscopy-Based Deep Convolutional Neural Network Predicts Response to Neoadjuvant Treatment for Locally Advanced Rectal Cancer
Source: Front Physiol. 2022 Apr 27;13:880981. doi: 10.3389/fphys.2022.880981 (PMC9091815; doi:10.3389/fphys.2022.880981)
Supplement: Supplementary file 1 [file DataSheet1.pdf]

Supplementary method section

Image preprocessing

All images were subjected to unified standardized processing before DCNN model training to reduce variations caused by different image-acquisition systems. In the first step, tumor areas/scar were defined by experienced endoscopists, and we removed the invalid background of each image, and converted the images into a square image of the same size; a representative sample is shown in Figure S1A. Next, a brightness-equalized algorithm was used to eliminate the influence of different lighting, and then, an edge-detection algorithm was used to fetch the edges of each image to obtain a grayscale image, which was combined with the original RGB-mode image to form a four-dimensional channel image (Figure S1B). After that, we entered the channel image into the DCNN model for feature extraction and classifier training. We used image-augmentation techniques, including rotation, left-right flip, Gaussian blur, stochastic Gaussian noise, luminance changes, gamma contrast, random displacement, random scaling, left-right tilt, up-down tilt, perspective transformation, and random discarding of pixels, to prevent overfitting. Adjusting the corresponding parameters of the above enhancement techniques and combining them in different arrangements could generate a large amount of new training data, which greatly increased the sample size and ensured that the model had good generalizability. A representative picture is shown in Figure S1C. After completing the above operations, we used ImageNet for model pre-training.

Supplementary result section

Univariate analysis and multivariate analysis of factors associated with TRG0

To identify the factors that correlated with tumor response, we performed univariate and multivariate analyses for LARC patients with complete clinicopathological data. Age, neoadjuvant radiotherapy, differentiation, and preoperative CEA level were identified as significant factors in the univariate analysis. Multivariate analysis revealed that patients who were less than 50 years old, had received neoadjuvant radiotherapy, had well-differentiated tumors, or had negative preoperative serum CEA levels (<5 μg/ml) were more likely to achieve TRG0 (Supplementary Table S1).

Supplementary Table S1. Risk factors of TRG0 in the training cohort and validation cohort.

	Total (n, %)	TRG0	non-TRG0	univariate analysis		multivariate analysis	
				χ^2	***P***	OR (95% CI)	***P***
Age/years				6.60	0.01*	0.63(0.45-0.89)	0.01*
≥50	654(70.3)	130(63.1%)	524(72.4%)				
<50	276(29.7)	76(36.9%)	200(27.6%)				
Sex				0.10	0.75		
male	669(71.9%)	150(72.8%)	519(71.7%)				
female	261(28.1%)	56(27.2%)	205(28.3%)				
BMI (Kg/m^2)				2.12	0.35		
18.5≤BMI < 24	524(56.3)	114(55.3%)	410(56.6%)				
BMI<18.5	78(8.4)	13(6.3%)	65(9.0%)				
BMI>24	328(35.3)	79(38.4%)	249(34.4%)				
Neoadjuvant radiotherapy				14.56	<0.01*	1.68(1.22-2.32)	<0.01*
yes	380(40.9%)	108(52.4%)	272(37.6%)				
no	550(59.1%)	98(47.6%)	452(62.4%)				
Differentiation				33.05	<0.01*		<0.01*
well	259(27.8%)	90(43.7%)	169(23.3%)			reference	
moderate	592(63.7%)	102(49.5%)	490(67.7%)			0.42(0.30-0.59)	<0.01*
poor	79(8.5%)	14(6.8%)	65(9.0%)			0.40(0.21-0.77)	<0.01*
Initial CEA (ng/ml)				0.25	0.62		
>5	407(43.8)	87(42.2%)	320(44.2%)				
≤5	523(56.2)	119(57.8%)	404(55.8%)				
Pre-CEA (ng/ml)				29.24	<0.01*	0.24(0.13-0.44)	<0.01*
>5	175(18.8)	12(5.8%)	163(22.5%)				
≤5	755(81.2)	194(94.2%)	561(77.5%)				

*Significant different

TRG: Tumor regression grade

Pre-CEA: Pretreatment carcinoembryonic antigen.

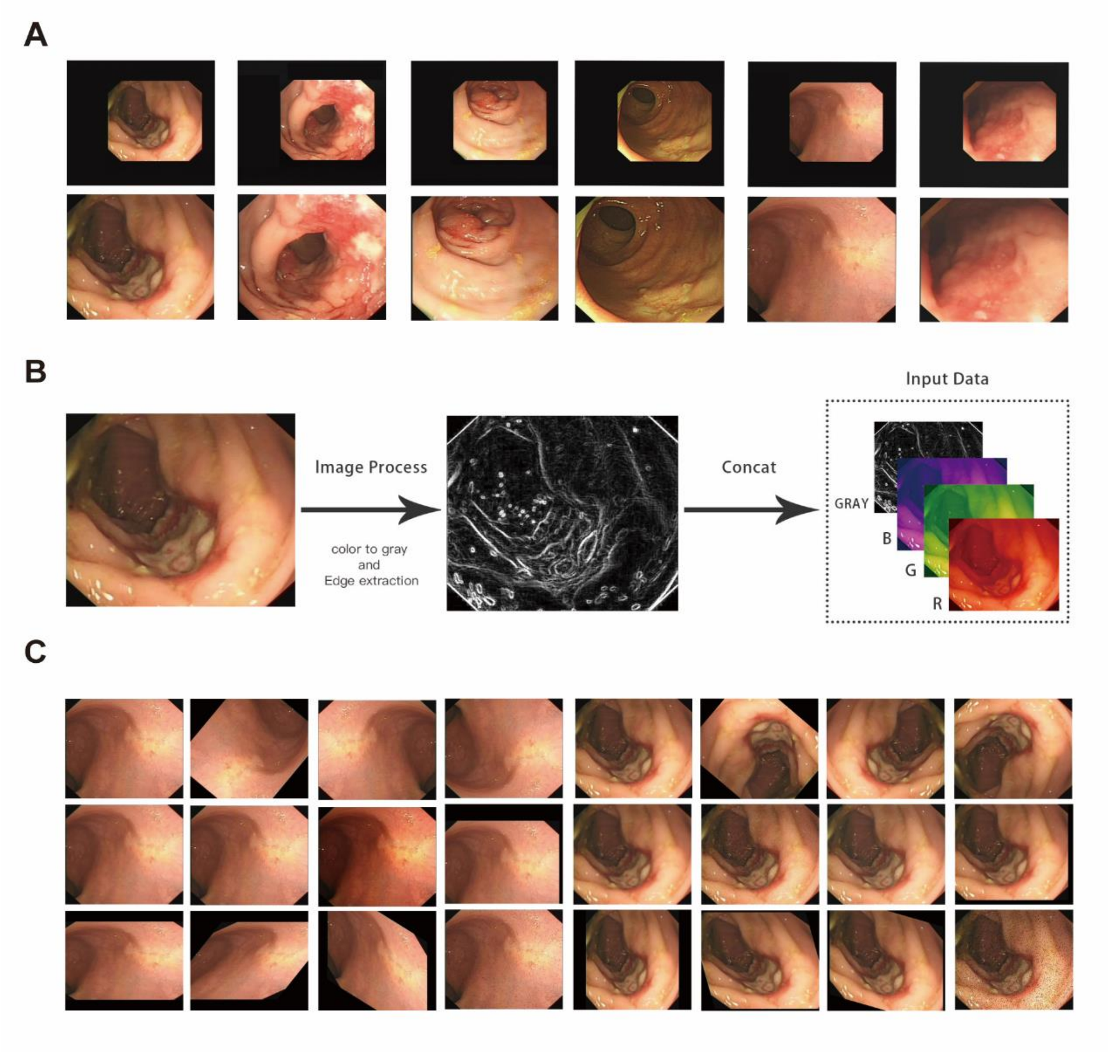

Figure S1. Representative images used for image preprocessing.

(A) Invalid background removed from the original images. (B) A four-channel image established from the combination of the original RGB-mode image with the grayscale image created by the edge-detection algorithm. (C) Image augmentation for a representative CR case (left) and a representative non-CR case (right). CR, complete response.
